# SERCA inhibition improves lifespan and healthspan in a chemical model of Parkinson disease in *Caenorhabditis elegans*

**DOI:** 10.3389/fphar.2023.1182428

**Published:** 2023-05-22

**Authors:** Silvia Romero-Sanz, Elena Caldero-Escudero, Pilar Álvarez-Illera, Jaime Santo-Domingo, Rosalba I. Fonteriz, Mayte Montero, Javier Álvarez

**Affiliations:** Departamento de Bioquímica y Biología Molecular y Fisiología, Facultad de Medicina, Unidad de Excelencia Instituto de Biomedicina y Genética Molecular (IBGM), Universidad de Valladolid y Consejo Superior de Investigaciones Científicas (CSIC), Valladolid, Spain

**Keywords:** *C. elegans*, rotenone, Parkinson’s disease, SERCA, lifespan, endoplasmic reticulum, mitochondria, Ca^2+^ signaling

## Abstract

**Introduction:** The high prevalence of neurodegenerative diseases in our population and the lack of effective treatments encourage the search for new therapeutic targets for these pathologies. We have recently described that submaximal inhibition of the Sarco-Endoplasmic Reticulum Ca^2+^ ATPase (SERCA), the main responsible for ER calcium storage, is able to increase lifespan in *Caenorhabditis elegans* worms by mechanisms involving mitochondrial metabolism and nutrient-sensitive pathways.

**Methods:** We have studied here the effects of submaximal SERCA inhibition in a chemical model of Parkinson’s disease (PD) induced in *C. elegans* worms by treatment with the mitochondrial complex I inhibitor rotenone. For specific SERCA inhibition, we treated worms with RNAi against *sca-1*, the sole orthologue of SERCA in *C. elegans*.

**Results and Discussion:** Our results show that rotenone produces alterations in worms that include decreased lifespan, smaller size, reduced fertility, decreased motility, defecation and pumping rate, increased mitochondrial ROS production, reduced mitochondrial membrane potential and oxygen consumption rate, altered mitochondrial structure, and altered ethanol preference in behavioral studies. Most of these alterations were either fully or partially reversed in worms treated with *sca-1* RNAi, suggesting that SERCA inhibition could be a novel pharmacological target in the prevention or treatment of neurodegeneration.

## 1 Introduction

Parkinson’s disease (PD) is a neurodegenerative disease characterized primarily by motor disturbances, including tremor, rigidity and slow movements. In addition, PD patients also show non-motor symptoms, such as sleep disturbances, dementia, and sensory or autonomic system abnormalities (Obeso et al., 2010; Poewe et al., 2017). Although approximately 10%–15% of PD cases are due to mutations in various genes (Deng et al., 2018), most cases are sporadic and their frequency increases with age, especially after 65 years. From the histopathological point of view, it is characterized by a loss of dopaminergic neurons in the substantia nigra, although it can also affect other areas of the central and peripheral nervous system, and at the cellular level it is characterized by the presence of Lewy bodies, inclusions composed mainly of the protein α-synuclein (Obeso et al., 2010; Poewe et al., 2017).

The origin of sporadic PD is not known, but there is evidence that environmental factors play a role, especially exposure to pesticides such as rotenone or paraquat, which may act in genetically predisposed individuals. In fact, the difficulty of generating good animal models of PD has led to the use of some of these pesticides to induce an analogous pathology in various types of animals (Greenamyre et al., 2010; Zeng et al., 2018; Thirugnanam and Santhakumar, 2022), including *C. elegans* (Zhou et al., 2013; Martinez et al., 2017; Wu et al., 2018; Zeng et al., 2018; Brunetti et al., 2020; Di Rosa et al., 2020; Gonzalez-Hunt et al., 2021; Zhang et al., 2021; Thirugnanam and Santhakumar, 2022).

One of the most widely used PD models employs the plant-based insecticide rotenone. Rotenone intoxication in rats generates a disease with many features characteristic of PD, including degeneration of nigrostriatal dopaminergic neurons, presence of α-synuclein inclusions similar to Lewy bodies in dopaminergic neurons, and motor deficits (Greenamyre et al., 2010). The mechanism by which rotenone acts to produce these effects is not fully elucidated. Rotenone is a high-affinity inhibitor of complex I of the respiratory chain, and produces a significant decrease in mitochondrial membrane potential in *C. elegans* and other animal models (Moon et al., 2005; Wu et al., 2018). However, at the doses used to mimic PD it does not alter oxygen consumption in isolated brain mitochondria, indicating that it does not generate a bioenergetic deficit (Sherer et al., 2003). Instead, there is evidence that rotenone toxicity may be mediated by oxidative stress arising as a consequence of respiratory chain complex I inhibition (Sherer et al., 2003; Sanders and Greenamyren, 2013). In fact, it has been shown in several cellular and animal models that rotenone causes an increase in ROS production (Sherer et al., 2003; Zeng et al., 2018; Vrijsen et al., 2020; Zhang et al., 2021) and both ROS production and lethality were reduced in a *C. elegans* strain by the mitochondria specific superoxide scavenger MitoTEMPO (Vrijsen et al., 2020).

There is evidence that alterations in Ca^2+^ homeostasis may play a relevant role in the neurodegeneration process responsible for PD (Zampese and Surmeier, 2020; Xu et al., 2022). We have recently described that some modulators of Ca^2+^ homeostasis, in particular inhibitors of the SERCA pump, are able at certain submaximal doses to increase the longevity of *C. elegans* worms (García-Casas et al., 2018; 2021a). The SERCA pump is the Ca^2+^ pump responsible for Ca^2+^ accumulation in the ER and is key for determining the level of Ca^2+^ in the ER and thus controlling cell activation processes involving Ca^2+^ release from the ER. Therefore, partial inhibition of SERCA should reduce the level of Ca^2+^ in the ER and modulate these processes (García-Casas et al., 2021a).

From these data, we hypothesized whether these inhibitors could also improve the phenotype of a PD model in *C. elegans*. In this work we show that SERCA inhibition with RNAi is indeed able to reverse some of the rotenone-induced alterations in the *C. elegans* model. Therefore, this ER Ca^2+^ pump could be a new pharmacological target to fight PD.

## 2 Materials and methods

### 2.1 *C. elegans* strains and maintenance

*C. elegans* nematodes were maintained and handled as previously described (Stiernagle, 2006). Strains were maintained at 20°C. The SJ4103 {zcls14 [myo-3GFP (mit)]}, wild-type strain that expresses GFP at high levels in mitochondria of body wall muscle cells, was obtained from the *Caenorhabditis* Genetics Center (CGC), which is funded by NIH Office of Research Infrastructure Programs (P40 OD010440).

### 2.2 Administration of rotenone and *sca-1* RNAi

Partial RNAi knock down of the SERCA pump was carried out as previously described (García-Casas et al., 2021a). The HT115 RNAi clone for *sca-1* (K11D9.2, Ahringer library) and empty vector (L4440) were kindly provided by Dr. Malene Hansen, Sanford Burnham Prebys Medical Discovery Institute, La Jolla, CA, United States. Nematode growth medium (NGM) agar plates containing 1 mM isopropyl-β-D-thiogalactoside (IPTG), 50 μM carbenicillin and 30 μM fluorodeoxyuridine, were seeded either with L4440 bacteria or with 90% L4440% and 10% *sca-1* RNAi bacteria. When appropriate, rotenone was then added over the agar to obtain the desired final concentration assuming homogeneous distribution. After that, young adult worms (day 1) were transferred onto the plates for the different assays. Therefore, worms were exposed to rotenone and *sca-1* RNAi at the same time on day 1 of adult life.

### 2.3 *C. elegans* lifespan assays

Lifespan assays were carried out as previously described (García-Casas et al., 2021a). Briefly, synchronized young adult worms (day 1) were transferred to NGM plates seeded with L4440 bacteria or 10% *sca-1* RNAi bacteria and 90% L4440 bacteria, and with or without rotenone, as required. Plates contained also 15 μM Fluorodeoxyuridine (FUdR) to avoid progeny (35 mm plates, 10 plates per condition with 10 worms/plate, at least 100 worms per condition). Control and drug-containing assays were always carried out in parallel in a temperature controlled incubator set at 20°C. Plates were scored for dead worms every day. Worms that did not respond to touch with a platinum wire were considered dead. Age refers to days following adulthood. Plates with fungal contamination during the first 10 days of the assay were excluded from the study. Missing worms, individuals with extruded gonad or desiccated by crawling in the edge of the plate were censored.

### 2.4 Tracking and mobility

Mobility assays were performed in N2 worms prepared in the different conditions as detailed above, at day 5 of adult life. One minute videos were taken in every condition, and they were analyzed offline using the ImageJ plugin “wrMTrck”, as described previously (Nussbaum-Krammer et al., 2015).

### 2.5 Defecation and pharyngeal pumping

Defecation was measured in N2 worms prepared in the different conditions as detailed above, at day 3 of adult life. 30 min videos were recorded and the number of defecations (measured as posterior body muscle contraction) during that time was then counted in at least 10 worms per condition. The rate of pharyngeal pumping was counted for 20 s every minute for 10 min in each worm. At least 10 different worms at day 3 of adult life were examined in each condition.

### 2.6 Mitochondrial ROS

Mitochondrial ROS were measured in N2 worms prepared in the different conditions as detailed above, at day 5 of adult life. 20–30 worms were collected, washed with M9 medium and incubated with shaking for 4.5 h in the dark with 500 µL of 10 µM MitoSOX in M9 medium. Worms were then washed with M9 medium, and 5-6 worms are placed on an agar pad with a drop of 50 mM sodium azide prior to confocal imaging of the pharynx region in a Leica TCS SP5 confocal microscope (excitation 510 nm, emission 580 nm). Fluorescence intensity was proportional to the level of mitochondrial ROS.

### 2.7 Mitochondrial membrane potential

Mitochondrial membrane potential was measured in N2 worms prepared in the different conditions as detailed above, at day 5 of adult life. 20–30 worms were collected, washed with M9 medium and incubated with shaking for 3.5 h in the dark with 500 µL of 0.1 µM TMRE in M9 medium. An additional control tube was prepared by adding 100 µM of the protonophore CCCP. Worms were then washed with M9 medium, and 5-6 worms are placed on an agar pad with a drop of 10 mM tetramisol prior to fluorescence imaging in a Nikon ECLIPSE Ni-E microscope equipped with a TRITC filter and a DS-Ri2camera. Fluorescence intensity was proportional to the mitochondrial membrane potential.

### 2.8 Oxygen consumption rate

We measured oxygen consumption in *C. elegans* as previously described (Koopman et al., 2016) using an XFe24 metabolic analyzer (Agilent, United States). Thirty minutes before the experiment, we washed day 5 synchronized adult worm populations twice with M9 medium and transferred them to a tissue culture plate (Seahorse, Agilent) at a density of 15–30 worms per well. Respiration rates were measured every 5 min using the following measurement protocol: 2 min mixing, 30 s waiting, and 2 min measuring. All experiments were performed at 25°C. Respiratory chain modulators were added as indicated in the figures at the following concentrations: 10 µM FCCP; 50 mM Sodium Azide.

### 2.9 Mitochondrial structure

For confocal imaging of mitochondrial structure, SJ4103 day 5 worms prepared in the different conditions detailed above were transferred to a 2% agarose pad containing a drop of 50 mM sodium azide to paralyze worms. A coverslip was placed on top and worms were imaged on a Leica TCS SP5 confocal microscope (excitation 488 nm, emission 500–554 nm). Images were analyzed using the ImageJ plug-in Mitochondria Analyzer (Chaudhry et al., 2020) to calculate several morphological parameters: aspect ratio, mitochondrial number per cell, form factor and number of branches per mitochondria.

### 2.10 Associative learning

Associative learning was studied as described previously (Lee et al., 2009). Briefly, we used N2 worms prepared in the different conditions as detailed above, at day 5 of adult life. Worms were then placed for 4 h in plates either with or without 300 mM Ethanol. Worms were then washed with M9 medium and placed in the center of plates divided into four quadrants, in two of which, diagonally across, a drop of 10 μL of 300 mM ethanol was placed. A photograph was taken then and 30 min afterword, and the preference index (PI) was calculated as PI = (worms in Ethanol quadrants—worms in control quadrants)/total worms (Lee et al., 2009).

### 2.11 Fertility

Fertility was measured in N2 worms prepared in the different conditions as detailed above, in the absence of FUdR. Worms were placed in 24-well plates, 1 worm per well in 6 wells. Worms were then changed to new wells every day, during 4 days, and then eggs and larvae in each well were counted.

### 2.12 Materials

Rotenone and CCCP (carbonyl cyanide 3-chlorophenylhydrazone) were obtained from Sigma, Madrid, Spain. TMRE (tetramethylrhodamine, ethyl ester) and MitoSOX Red were obtained from Molecular Probes (Thermo Fisher Scientific). FuDR was acquired from Alfa Aesar, Karlsruhe, Germany. Other reagents were from Sigma, Madrid, Spain or Merck, Darmstadt, Germany.

### 2.13 Statistical analysis

In the lifespan assays, statistical analysis was performed with the SPSS software, using the Kaplan-Meier estimator and the log-rank routine for significance of each assay. Three assays per condition were made, and the half-lives were compared using ANOVA and Tukey’s test. The significance of each assay is shown in Supplementary Table S1. In the rest of the studies, data are shown as mean ± s.e.m. and significance was calculated using ANOVA test and *post hoc* comparisons made with Tukey’s test. *, *p* < 0.05; **, *p* < 0.01; ***, *p* < 0.005.

## 3 Results

### 3.1 Effects on lifespan

**FIGURE 1:**
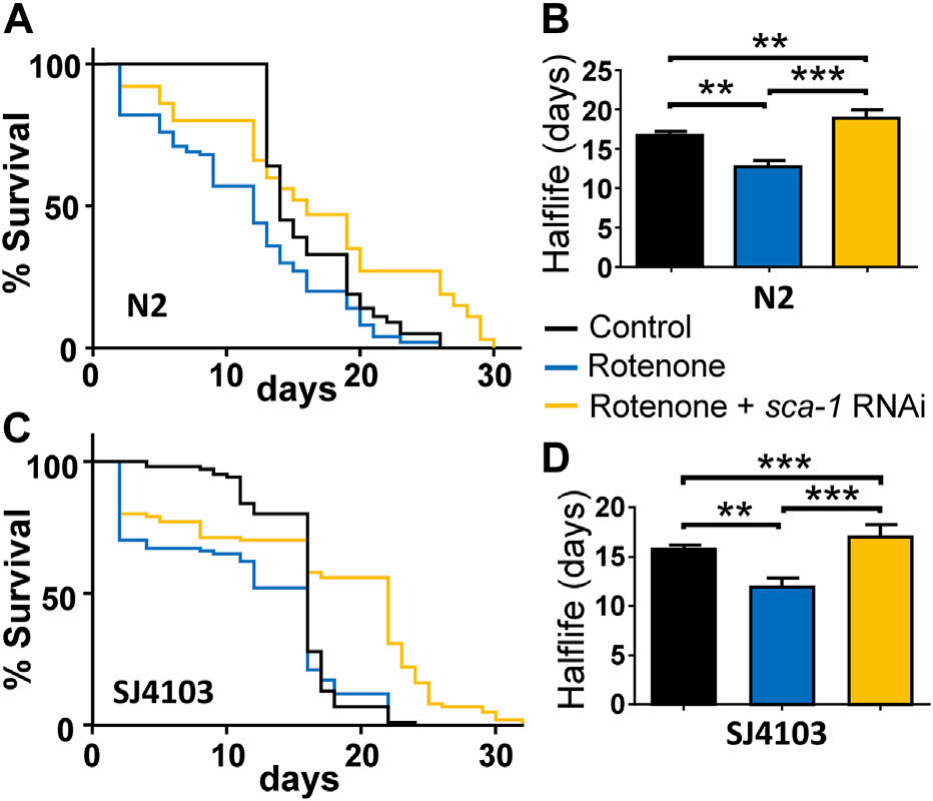
Rescue by *sca-1* RNAi of the effect of rotenone on *C. elegans* lifespan. Panels **(A,C)** show survival curves obtained in the N2 and SJ4103 strains in either control worms or worms treated with rotenone or rotenone and 10% *sca-1* RNAi. SJ4103 is an N2 strain expressing a mitochondrial GFP in body wall muscle cells. Panels **(B,D)** show the mean half-life obtained in 3 similar assays of each kind (see Supplementary Table S1). Rotenone concentration was 14 µM in the N2 strain and 5 µM in the SJ4103 strain. Data in panels **(B,D)** are mean ± s.e.m. **, *p* < 0.01; ***, *p* < 0.005.

Treatment of wild-type *C. elegans* nematodes with rotenone produces a decrease in their half-life composed of two phases. There is an initial phase of high mortality that appears immediately after the addition of the drug, but worms that survive this phase exhibit an apparently normal survival curve, albeit with a decreased half-life compared to that of controls (Figure 1).

The magnitude of the first phase of initial mortality was proportional to the dose of rotenone, and for that reason we chose for our study a dose capable of generating reduced mortality in that first phase, in order to be able to assess the effect of rotenone and its possible reversal by partial inhibition of SERCA in the remaining worms. For SERCA inhibition we used RNAi against the *sca-1* gene, which encodes for the only homologue of the SERCA pump in *C. elegans*. Moreover, this RNAi is used at 10% dilution to generate only partial inhibition. We have previously reported that this dilution, that reduces SCA-1 mRNA expression to 27% ± 2% of the control, is the most effective to increase lifespan in N2 wild-type worms (García-Casas et al., 2021a). We obtained similar effects with SERCA inhibitors such as thapsigargin and 2, 5-BHQ, which also increased *C. elegans* lifespan only at submaximal concentrations (García-Casas et al., 2018).

Figure 1A shows a lifespan assay comparing the survival of wild-type N2 strain either alone, in the presence of 14 µM rotenone or in the presence of 14 µM rotenone and treated with 10% RNAi against the *sca-1* gene. Worms were exposed simultaneously to both rotenone and *sca-1* RNAi on day 1 of adult life. It can be seen that rotenone produces a small initial drop in survival (17.0% ± 2.6%, mean ± sem, *n* = 3), and the rest of the treated worms follow a mortality curve with a half-life about 20% smaller with respect to the control (Supplementary Table S1). Treatment with 10% *sca-1* RNAi changed little of the initial toxicity (11.7% ± 2.7%, mean ± sem, *n* = 3), but significantly lengthened the half-life of the remaining worms, which in this assay was even longer than that of the control worms (Figure 1B). In the Supplementary Material; Supplementary Table S1 shows details of number of worms, half-life and significance from three similar survival assays leading to the same conclusion.

We have performed similar experiments using as a control another *C. elegans* strain derived from the N2 wild-type strain, SJ4103, which expresses GFP inside body wall muscle mitochondria. We will use this strain later to study mitochondrial morphology. We obtained the same results, although this strain turned out to be somewhat more sensitive to the initial toxic effect of rotenone and the dose had to be reduced to 5 µM. Figures 1C, D show the effects of rotenone and *sca-1* RNAi in this strain, and Supplementary Table S1 shows the data from 3 lifespan assays made. Initial drop in survival in this strain was 31.0% ± 3.2% in the presence of rotenone and 27.3% ± 4.3% in the presence of rotenone + *sca-1* RNAi (mean ± sem, *n* = 3).

### 3.2 Effects on muscle activity: mobility, defecation and pharyngeal pumping

**FIGURE 2:**
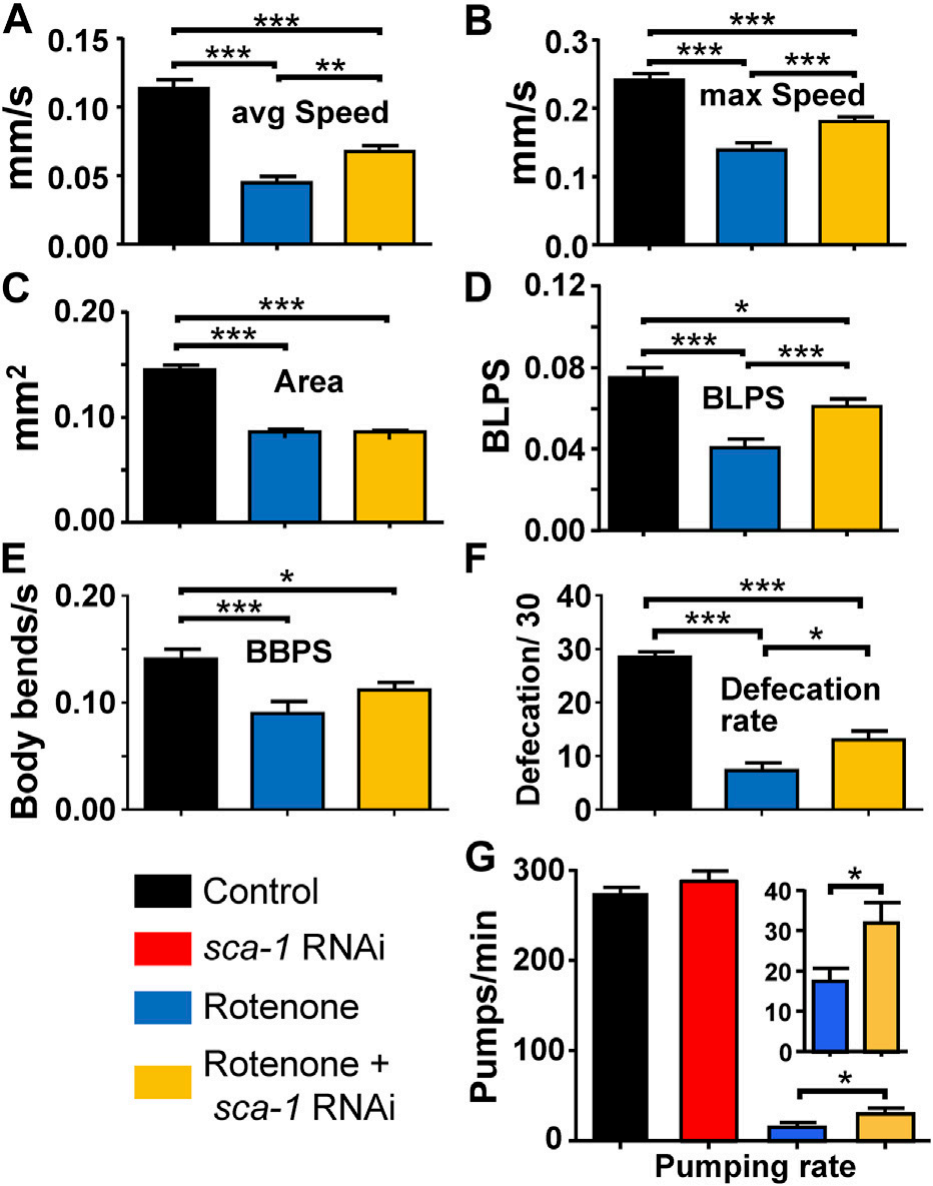
Rescue by *sca-1* RNAi of the effect of rotenone on N2 *C. elegans* mobility and defecation. The figure shows the average speed (panel **(A)**), maximum speed (panel **(B)**), size (panel **(C)**), speed measured as body length per second (BLPS, panel **(D)**, body bends per second (panel **(E)**), rate of defecation (panel **(F)**) and rate of pumping (panel **(G)**) in either control worms or worms treated with rotenone or rotenone and 10% *sca-1* RNAi. Data are mean ± s.e.m. *, *p* < 0.05; ***, *p* < 0.005.

To investigate whether the reversal of the effect of rotenone by SERCA inhibition also resulted in an improvement of healthspan, we performed several functional studies. First, we measured worm mobility under the different conditions. The measurements were performed on day 5 adult N2 worms, previously treated or not with rotenone or rotenone + *sca-1* RNAi from day 1. Figures 2A, B shows that the average and maximum speed of worms was significantly reduced in rotenone-treated nematodes, and SERCA inhibition partially reversed this effect in both cases. In these experiments we also found that rotenone produced a marked decrease in worm size, measured as worm area, of almost 50% (Figure 2C). This decrease was not rescued by treatment with *sca-1* RNAi. The smaller size of the worms may also affect their speed, and to compensate for this factor we also measured speed in terms relative to the size of the body, that is, in units of body length per second (BLPS). Using this measurement the effect of rotenone decreasing velocity was maintained and the reversal of the effect by *sca-1* RNAi was found to be the same (Figure 2D). As *C. elegans* nematodes move by bending their body in sinusoidal waves, we have also measured the number of body bends per second (BBPS) as a parameter of good mobility. BBPS also decreased in the presence of rotenone and this effect was partially reversed in the presence of *sca-1* RNAi (Figure 2E). Supplementary Table S2 shows the mean data and number of worms studied in each case.

We also tested the effect of *sca-1* RNAi on the mobility of wild-type N2 worms and found no effect on any parameter (see Supplementary Material; Supplementary Figure S1). This suggests that the reversal of rotenone-induced mobility impairment we found is specific to rotenone toxicity.

Another method to measure muscle activity in worms is defecation. We measured the number of defecations performed in 30 min in day 3 adult worms in the three conditions, wild type and pretreated with rotenone or rotenone + *sca-1* RNAi from day 1. Day 3 was chosen in this case because the rate of defecation decreases considerably afterwards. As with motility, rotenone treatment reduced the number of defecations, and SERCA inhibition partially reversed the effect (Figure 2F). To investigate whether these effects on defecation could be due to interference with feeding, we also measured the rates of pharyngeal pumping in each condition. The results show that the rate of pharyngeal pumping was much lower in the presence of rotenone. This may explain, at least in part, the decrease in the defecation rate. Treatment with *sca-1* RNAi did not alter pharyngeal pumping in the controls, but resulted in a small but significant increase in the pumping rate after rotenone treatment. Thus, rotenone increased the overall length of the intestinal cycle and *sca-1* RNAi partially restored it.

### 3.3 Effects on mitochondrial function: ROS, membrane potential and oxygen consumption rate

**FIGURE 3:**
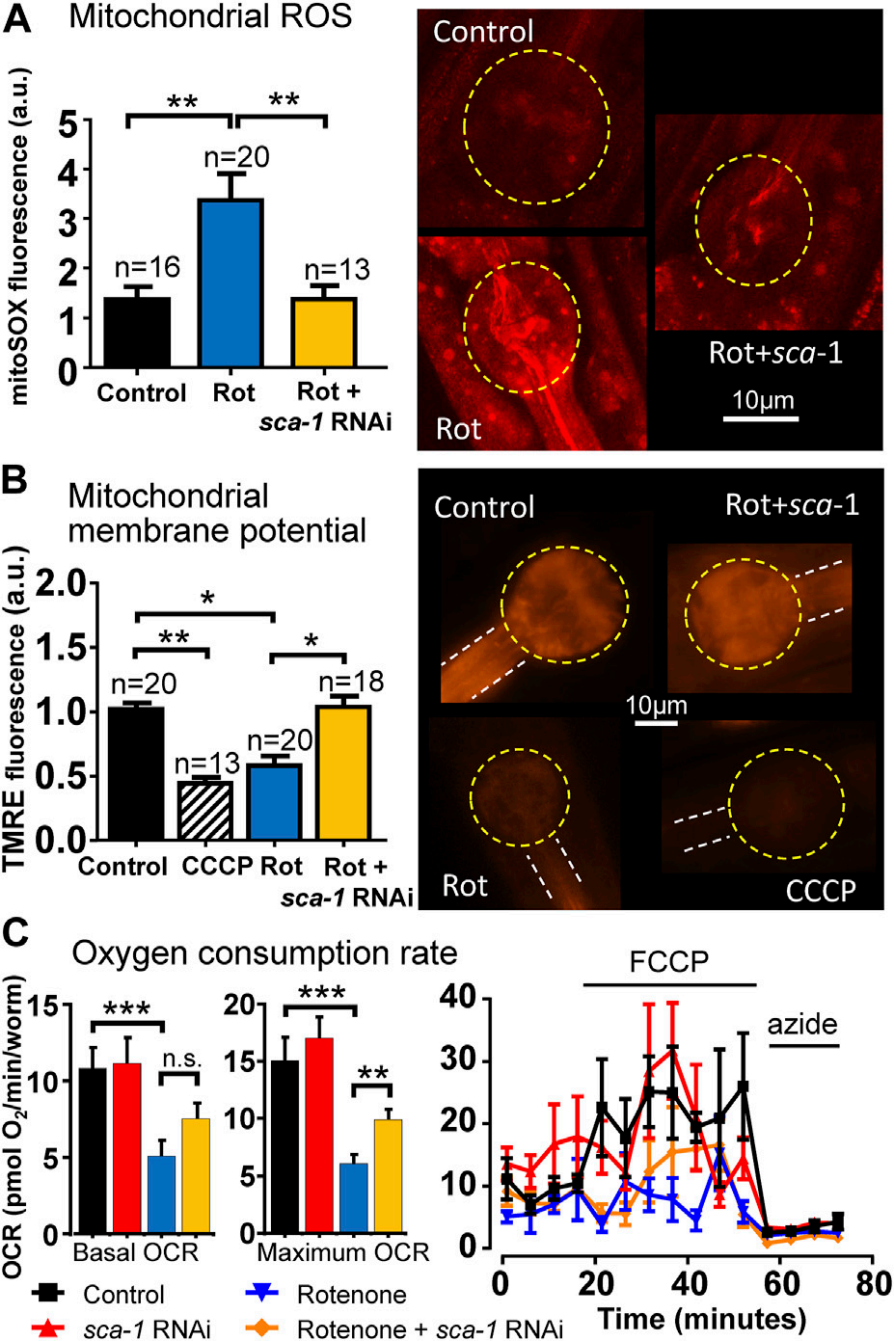
Rescue by *sca-1* RNAi of the effect of rotenone on mitochondrial ROS production, mitochondrial membrane potential and oxygen consumption rate (OCR) in N2 *C. elegans*. Panel **(A)** shows mitochondrial ROS measurements with mitoSOX, panel **(B)** shows mitochondrial membrane potential measurements with TMRE and panel **(C)** shows OCR measurements, all of them obtained either in control worms or in worms treated with rotenone or rotenone and 10% *sca-1* RNAi. The protonophore CCCP was also included to induce full mitochondrial membrane depolarization in panel **(B)**. In OCR measurements, the protonophore FCCP was added to obtain the maximum rate of respiration, and azide to obtain non-mitochondrial respiration. The number of experiments made is included on top of each bar in panels **(A,B)**. For OCR measurements, 12 experiments were made in three different days. Representative images of fluorescence of each kind are shown in the right part of the figure in panels **(A,B)**. Data are mean ± s.e.m. *, *p* < 0.05; **, *p* < 0.01; ***, *p* < 0.005.

As we mention above, rotenone leads to increased ROS production as a consequence of inhibition of complex I of the respiratory chain. If the toxic effects of rotenone are mediated in part by ROS formation, it would be important to test whether SERCA inhibition reverses ROS formation. Figure 3A shows that this is the case. Addition of rotenone produced a significant increase in mitochondrial ROS production measured with mitoSOX red and inhibition of SERCA with *sca-1* RNAi completely reversed this effect.

We have also mentioned above that rotenone produces a significant decrease in mitochondrial membrane potential. We have therefore studied whether SERCA inhibition is able to reverse this effect. To measure mitochondrial membrane potential, we used TMRE, a positively charged cell permeant dye, which readily accumulates in mitochondria due to the negative electrical potential of the matrix. As can be seen in Figure 3B, rotenone produced a large decrease in mitochondrial membrane potential, measured with the fluorescent dye TMRE, close to that produced by the protonophore CCCP, which abolishes the mitochondrial membrane potential and results in the release of TMRE into the cytosol. And here again inhibition of SERCA with *sca-1* RNAi restored the membrane potential to the value of the controls.

Another important parameter of mitochondrial function is oxygen consumption rate (OCR). We have measured here the effect of rotenone and rotenone + *sca-1* RNAi on *C. elegans* OCR measured with the Seahorse. Our data show that rotenone reduced both basal and maximum OCR, as would be expected for a mitochondrial complex I inhibitor, and this effect was partially rescued by SERCA inhibition with *sca-1* RNAi (Figure 3C). Non-mitochondrial respiration (that obtained in the presence of azide) was not modified.

### 3.4 Effects on mitochondrial structure

The mitochondrial structure of the body wall muscle degenerates with worm age in parallel with the development of sarcopenia and reduction in mobility (Gaffney et al., 2018). It is therefore another parameter of good muscle health. We have studied here the effect of rotenone and SERCA inhibition on mitochondrial structure measured from fluorescence images obtained in the strain SJ4103, which expresses GFP inside body wall muscle mitochondria.

**FIGURE 4:**
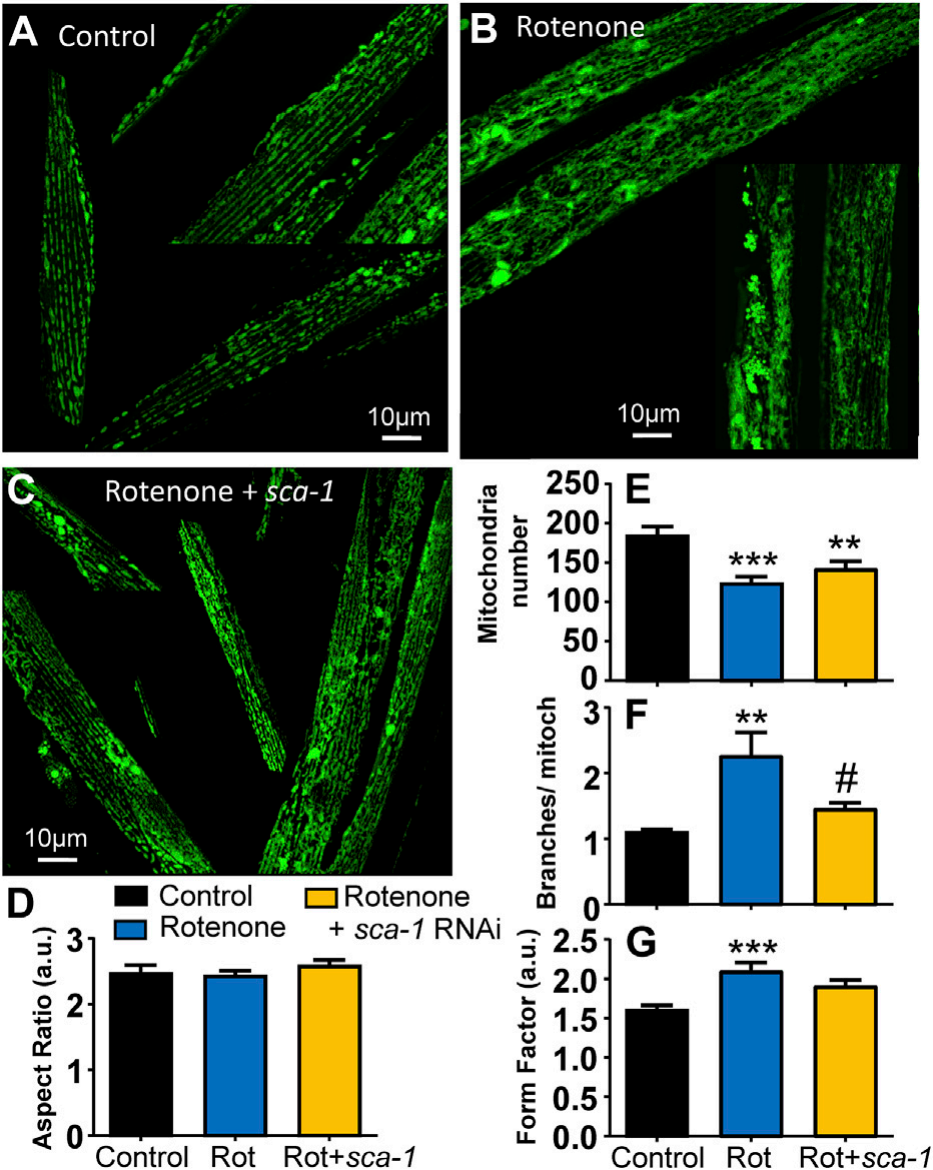
Rescue by *sca-1* RNAi of the effect of rotenone on mitochondrial structure in the SJ4103 strain expressing mitochondrial GFP. A series of representative confocal images of mitochondrial fluorescence are shown in the three different conditions: control worms (n = 17, panel **(A)**), worms treated with rotenone (n = 27, panel **(B)**) or rotenone and 10% *sca-1* RNAi (n = 25, panel **(C)**). Bar plots show the morphological analysis of the images obtained in each condition: aspect ratio (panel **(D)**), mitochondrial number per cell (panel **(E)**), the number of branches per mitochondria (panel **(F)**) and the form factor (panel **(G)**). Rotenone concentration was 5 μM **, *p* < 0.01 vs. Control; ***, *p* < 0.005 vs. Control; #, *p* < 0.05 vs. Rotenone.

Figure 4A shows a series of fluorescence images of control SJ4103 worms of day 5 of adult life, in which an ordered pattern of mitochondrial structure is visible. In contrast, a clear increase in the number of mitochondrial aggregates and a disorganization of mitochondrial structure is observed in worms treated with rotenone from day 1 (Figure 4B). SERCA inhibition with *sca-1* RNAi did not make the aggregates disappear, but the images suggest a better maintenance of parallel fiber organization (Figure 4C). To confirm this, the images obtained in the three conditions were analyzed with the ImageJ plug-in Mitochondria Analyzer (Chaudhry et al., 2020) to calculate morphological parameters that could evidence these changes. The results are shown in Figures 4D–G. There was no change in the aspect ratio (the width/height relationship), but we observed significant changes in the mitochondrial number per cell, the form factor and the number of branches per mitochondria. The mitochondrial number per cell decreased in the presence of rotenone, and they had more branches. Both effects were partially restored by SERCA inhibition. Consistently, the form factor, which reflects the complexity and branching aspect of mitochondria, increased in the presence of rotenone, and this effect was also partially rescued by SERCA inhibition.

### 3.5 Effects on associative learning

We have also studied the effect of the treatments on the response of *C. elegans* to the presence of ethanol and the adaptation of this response after pre-exposure to ethanol. It has been previously described that these worms show basal aversive responses to ethanol, but after a 4 h pre-exposure to ethanol the animals show instead a preference for ethanol. This behavior has been proposed as a model of complex associative learning (Lee et al., 2009). Furthermore, mutants deficient in dopamine synthesis were not able to develop this preference for ethanol (Lee et al., 2009), an effect that would also be expected in a PD model if there is damage in the dopaminergic neurons. Therefore, we thought it would be interesting to test whether this mechanism was disrupted by rotenone toxicity and restored by SERCA inhibition.

**FIGURE 5:**
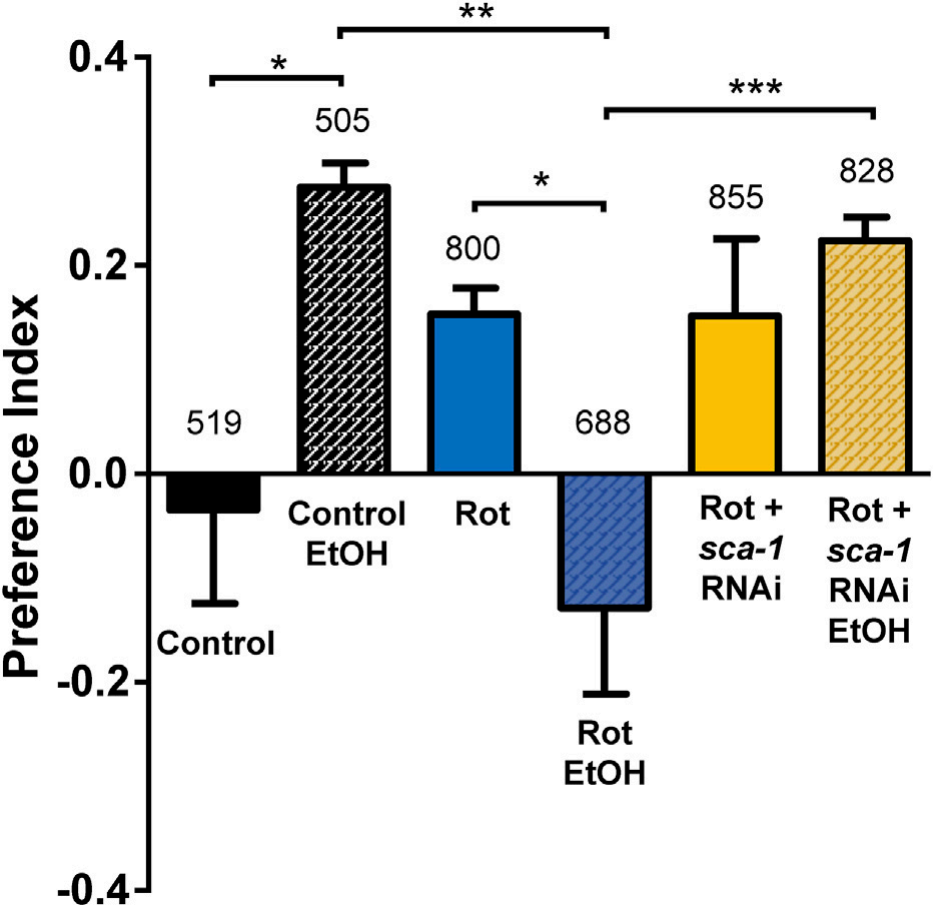
Rescue by *sca-1* RNAi of the effect of rotenone on ethanol preference. The figure shows the ethanol preference index obtained in the three different conditions: control N2 worms or N2 worms treated with rotenone or rotenone and 10% *sca-1* RNAi. Data are mean ± s.e.m. of 5 different experiments of each kind. *, *p* < 0.05; **, *p* < 0.01; ***, *p* < 0.005. Rotenone concentration was 14 µM. The number of worms tested is above each bar.

The results of our experiments are shown in Figure 5. We calculated the preference index in each of our conditions as described earlier (Lee et al., 2009), and the results were very interesting. As previously described, the basal preference index in the wild type strain is negative, but becomes positive after pre-exposure to ethanol. In contrast, the results in the rotenone-exposed strain are opposite. Under basal conditions the rotenone-treated worms have a preference for ethanol, but after pre-exposure to ethanol they show a clear aversion to ethanol. Finally, treatment with *sca-1* RNAi does not modify the effect of rotenone under basal conditions, i.e., they still prefer ethanol, but the preference changes completely after pre-exposure to ethanol. In this case, the worms show a strong preference for ethanol after ethanol pre-exposure, the same behavior observed in the controls. Therefore, ethanol clearly disrupts associative learning of ethanol preference, and SERCA inhibition produces also here a partial reversal of the alteration.

### 3.6 Effects on fertility

The effect of rotenone and *sca-1* RNAi on fertility was also evaluated. Rotenone added at day 1 of adult life produced a huge decrease in fertility, to values not exceeding 5% of controls. In this case, inhibition of SERCA with *sca-1* RNAi did not produce any significant improvement in fertility (Supplementary Figure S2).

## 4 Discussion

In our laboratory we have recently found that modulation of some systems involved in intracellular Ca^2+^ homeostasis is capable of extending lifespan and improving functional parameters representative of better health in *C. elegans* nematodes (García-Casas et al., 2018; 2019; 2021a; 2021b). One of these systems is the ER Ca^2+^ pump or SERCA, whose partial inhibition, either with inhibitors or with RNAi, produced a significant increase in the lifespan in wild type worms (García-Casas et al., 2018; 2021a). From experiments made with mutants of different pathways, the mechanism of this effect was found to be unrelated with dietary restriction, insulin signaling pathway or sirtuin activity, but required functional mitochondrial respiratory chain and both the AMP kinase and TOR pathways (García-Casas et al., 2018; 2021a).

In this work we have used a chemical model of Parkinson’s disease in *C. elegans* nematodes to study the effect of partial inhibition of the ER Ca^2+^ pump (SERCA) and its capacity to reverse the alterations of the model. As a disease-inducing agent we used the insecticide rotenone, a known inhibitor of complex I of the mitochondrial respiratory chain that generates PD-like alterations in several animal species (Greenamyre et al., 2010; Zeng et al., 2018; Thirugnanam and Santhakumar, 2022), and has been previously used as a model of PD in *C. elegans* (Zhou et al., 2013; Martinez et al., 2017; Wu et al., 2018; Zeng et al., 2018; Brunetti et al., 2020; Di Rosa et al., 2020; Gonzalez-Hunt et al., 2021; Zhang et al., 2021; Thirugnanam and Santhakumar, 2022).

Survival studies performed at various concentrations of rotenone showed that a dose of 14 µM produced a decrease in half-life of around 20% and an increase in initial mortality of no more than 20% in the N2 strain, and this dose was used in the rest of the studies. It is interesting to note that another closely similar strain, SJ4103, which differs only in the expression of a fluorescent sensor in body wall muscle mitochondria, showed a somewhat higher sensitivity to rotenone toxicity, so that we had to reduce the concentration of rotenone in this strain to 5 µM. The reason for this differential rotenone sensitivity is unclear.

Our data show that rotenone produces a decrease in worm survival, which is mainly accompanied by muscular and mitochondrial alterations. The muscular alterations are manifested in the form of a decrease in mobility, both in average and maximum speed and in the number of body bends generated during movement. There is also a large decrease in size, which is about halved, but this decrease is not responsible for the lower velocity, as the decrease in velocity is still observed when it is calculated in terms of body length per second (BLPS). Muscle defects also cause a significant decrease in the rate of defecation, which may be due in part to a reduction in the rate of pharyngeal pumping. Regarding mitochondrial alterations, rotenone was observed to produce an almost 3-fold increase in mitochondrial ROS generation and a decrease in membrane potential, in both cases measured at the pharynx by specific fluorescent probes. In addition, rotenone produced a disorganization of the mitochondrial structure, generating numerous well visible aggregates in worms expressing mitochondrial GFP in body wall muscle.

Most of these alterations were at least partially reversed after submaximal inhibition of SERCA with specific RNAi against the SCA-1 gene, the only SERCA representative in *C. elegans*. Rotenone reduced the half-life of the worms by about 20% and treatment with submaximal *sca-1* RNAi restored it to values similar to those of the control. In the case of motility, rotenone reduced all the motility parameters studied: average and maximum speed, body length per second and body bends per second, and treatment with *sca-1* RNAi partially restored the speed of movement. In the case of pharyngeal pumping and defecation rates, which are another manifestation of muscle activity, rotenone largely reduced both, and treatment with *sca-1* RNAi nearly doubled both of them, also partially reversing the effect.

Regarding mitochondrial activity, rotenone produced a significant increase in ROS production and treatment with *sca-1* RNAi returned the ROS level to control values. The same effect could be observed when studying mitochondrial membrane potential and OCR. Rotenone reduced the mitochondrial membrane potential almost as much as the protonophore CCCP and treatment with *sca-1* RNAi restored the membrane potential to control values. Similarly, rotenone reduced basal and maximum OCR and SERCA inhibition with *sca-1* RNAi also partially rescued this effect. By studying mitochondrial morphology in body wall muscle we were also able to verify that rotenone disrupts the regular pattern of mitochondrial structure and produces aggregates, whereas treatment with *sca-1* RNAi allows better preservation of mitochondrial structure, with partial restoration of the alterations induced by rotenone in mitochondrial number and morphology.

Finally, we have also conducted a behavioral assay studying the ethanol preference of the worms. It has been described that worms show an aversive reaction to ethanol under basal conditions, but instead show a preference for ethanol after 4 h of ethanol exposure (Lee et al., 2009). Furthermore, this response is altered in mutants defective in dopamine and serotonin synthesis (Lee et al., 2009), as might be expected in our worms treated with rotenone. Our data show that rotenone did indeed alter the response, such that treated worms showed ethanol preference under basal conditions and ethanol aversion after 4 h exposure to ethanol. Treatment with *sca-1* RNAi did not alter ethanol preference under basal conditions, but instead caused worms to maintain that preference after ethanol pre-exposure, as do controls. The mechanism of this partial reversal of the effect is unclear.

In summary, partial inhibition of SERCA is not only able to increase lifespan and healthspan in wild-type *C. elegans* worms, but is also able to fully or partially rescue a chemical PD model generated in these nematodes by rotenone treatment. The mechanism of this effect is probably a consequence of the decrease in the Ca^2+^ level in the ER following partial SERCA knockdown. This would condition a decrease in the amount of Ca^2+^ released during cell stimulation, either through the inositol 1, 4, 5-trisphosphate receptor (IP_3_R) or through the ryanodine receptor. This Ca^2+^ release is very important to activate some Ca^2+^-dependent phenomena during cell activation, one of them mitochondrial metabolism (Jouaville et al., 1999; Rossi et al., 2019). In fact, ER and mitochondria are connected by close contacts known as mitochondria-associated ER membranes (MAMs), and these contacts have been proposed to be involved in the progression of many diseases, including diabetes, cancer, cardiovascular and neurodegenerative diseases or aging (Gómez-Suaga et al., 2018; Kerkhofs et al., 2018; Moltedo et al., 2019; Gao et al., 2020; Barazzuol et al., 2021; Markovinovic et al., 2022). From the point of view of Ca^2+^ signaling, one of the most important functions of MAMs is the transfer of Ca^2+^ from IP_3_R in the ER to mitochondrial calcium uniporter channels in mitochondria (Moltedo et al., 2019; Markovinovic et al., 2022). This transfer should be significantly reduced after SERCA inhibition. As we have previously proposed (García-Casas et al., 2021a), this effect may reduce mitochondrial metabolism and ROS production, while at the same time activate systems that favor survival, including AMP-dependent kinase. By reducing mitochondrial damage, this mechanism may also be responsible for improved mitochondrial structure and functionality, including partial restoration of mitochondrial membrane potential and OCR. These phenomena may underlie the beneficial effects we found here in our PD model.

SERCA pump modulators could therefore be useful in the treatment of this neurodegenerative disease. Pharmacological targeting of SERCA has been proposed before. The SERCA pump plays a central and key role in cellular Ca^2+^ homeostasis, and has been considered early on as a possible target for pharmacological treatment in several pathological conditions. In fact, SERCA inhibition was proposed more than 20 years ago as a possible strategy for cardioprotection, in particular during ischemia-reperfusion (Zucchi et al., 2001). More recently, both SERCA activation and inhibition have been investigated as potential therapeutic tools. SERCA activation has attracted attention for the treatment of skeletal muscle diseases (Xu and Van Remmen, 2021), and SERCA inhibition has been studied mainly in the context of cancer therapy, where several clinical trials have been performed or are ongoing (Tadini-Buoninsegni et al., 2018; Peterková et al., 2020). These studies have stimulated the interest in finding new SERCA activators and inhibitors, the number of which is rapidly increasing (Tadini-Buoninsegni et al., 2018; Peterková et al., 2020; Pagliaro et al., 2021). In this paper we propose a new therapeutic target to which these compounds could be applied.

## Data Availability

The original contributions presented in the study are included in the article/Supplementary Material, further inquiries can be directed to the corresponding author.

## References

[B1] Barazzuol L. Giamogante F. Calì T. (2021). Mitochondria associated membranes (MAMs): Architecture and physiopathological role. Cell Calcium 94, 102343. 10.1016/J.CECA.2020.102343 33418313

[B2] Brunetti G. Di Rosa G. Scuto M. Leri M. Stefani M. Schmitz-Linneweber C. (2020). Healthspan maintenance and prevention of Parkinson’s-like phenotypes with hydroxytyrosol and oleuropein aglycone in *C. elegans* . Int. J. Mol. Sci. 21, 2588. 10.3390/IJMS21072588 32276415 PMC7178172

[B3] Chaudhry A. Shi R. Luciani D. S. (2020). A pipeline for multidimensional confocal analysis of mitochondrial morphology, function, and dynamics in pancreatic β-cells. Am. J. Physiol. - Endocrinol. Metab. 318, E87–E101. 10.1152/AJPENDO.00457.2019 31846372 PMC7052579

[B4] Deng H. Wang P. Jankovic J. (2018). The genetics of Parkinson disease. Ageing Res. Rev. 42, 72–85. 10.1016/J.ARR.2017.12.007 29288112

[B5] Di Rosa G. Brunetti G. Scuto M. Salinaro A. T. Calabrese E. J. Crea R. (2020). Healthspan enhancement by olive polyphenols in *C. elegans* wild type and Parkinson’s models. Int. J. Mol. Sci. 21, 3893. 10.3390/IJMS21113893 32486023 PMC7312680

[B6] Gaffney C. J. Pollard A. Barratt T. F. Constantin-Teodosiu D. Greenhaff P. L. Szewczyk N. J. (2018). Greater loss of mitochondrial function with ageing is associated with earlier onset of sarcopenia in *C. elegans* . Aging (Albany. NY) 10, 3382–3396. 10.18632/aging.101654 30455409 PMC6286836

[B7] Gao P. Yan Z. Zhu Z. (2020). Mitochondria-associated endoplasmic reticulum membranes in cardiovascular diseases. Front. Cell Dev. Biol. 8, 604240. 10.3389/FCELL.2020.604240 33240899 PMC7680862

[B8] García-Casas P. Alvarez-Illera P. Fonteriz R. I. Montero M. Alvarez J. (2021a). Mechanism of the lifespan extension induced by submaximal SERCA inhibition in *C. elegans* . Mech. Ageing Dev. 196, 111474. 10.1016/J.MAD.2021.111474 33766744

[B9] García-Casas P. Alvarez-Illera P. Gómez-Orte E. Cabello J. Fonteriz R. I. Montero M. (2021b). The mitochondrial Na+/Ca2+ exchanger inhibitor CGP37157 preserves muscle structure and function to increase lifespan and healthspan in *Caenorhabditis elegans* . Front. Pharmacol. 12, 695687. 10.3389/FPHAR.2021.695687 34211399 PMC8241105

[B10] García-Casas P. Arias-del-Val J. Alvarez-Illera P. Fonteriz R. I. Montero M. Alvarez J. (2018). Inhibition of sarco-endoplasmic reticulum Ca2+ ATPase extends the lifespan in *C. elegans* worms. Front. Pharmacol. 9, 669. 10.3389/FPHAR.2018.00669 29988547 PMC6026643

[B11] García-Casas P. Arias-Del-Val J. Alvarez-Illera P. Wojnicz A. De Los Ríos C. Fonteriz R. I. (2019). The neuroprotector benzothiazepine CGP37157 extends lifespan in *C. elegans* worms. Front. Aging Neurosci. 10, 440. 10.3389/FNAGI.2018.00440 30705628 PMC6344432

[B12] Gómez-Suaga P. Bravo-San Pedro J. M. González-Polo R. A. Fuentes J. M. Niso-Santano M. (2018). ER-mitochondria signaling in Parkinson’s disease. Cell Death Dis. 9, 337. 10.1038/s41419-017-0079-3 29497039 PMC5832754

[B13] Gonzalez-Hunt C. P. Luz A. L. Ryde I. T. Turner E. A. Ilkayeva O. R. Bhatt D. P. (2021). Multiple metabolic changes mediate the response of *Caenorhabditis elegans* to the complex I inhibitor rotenone. Toxicology 447, 152630. 10.1016/J.TOX.2020.152630 33188857 PMC7750303

[B14] Greenamyre J. T. Cannon J. R. Drolet R. Mastroberardino P. G. (2010). Lessons from the rotenone model of Parkinson’s disease. Trends Pharmacol. Sci. 31, 141–142. 10.1016/J.TIPS.2009.12.006 20096940 PMC2846992

[B15] Jouaville L. S. Pinton P. Bastianutto C. Rutter G. A. Rizzuto R. (1999). Regulation of mitochondrial ATP synthesis by calcium: Evidence for a long-term metabolic priming. Proc. Natl. Acad. Sci. U. S. A. 96, 13807–13812. 10.1073/PNAS.96.24.13807 10570154 PMC24146

[B16] Kerkhofs M. Bittremieux M. Morciano G. Giorgi C. Pinton P. Parys J. B. (2018). Emerging molecular mechanisms in chemotherapy: Ca2+ signaling at the mitochondria-associated endoplasmic reticulum membranes. Cell Death Dis. 9, 334. 10.1038/s41419-017-0179-0 29491433 PMC5832420

[B17] Koopman M. Michels H. Dancy B. M. Kamble R. Mouchiroud L. Auwerx J. (2016). A screening-based platform for the assessment of cellular respiration in *Caenorhabditis elegans* . Nat. Protoc. 11, 1798–1816. 10.1038/NPROT.2016.106 27583642 PMC5040492

[B18] Lee J. Jee C. McIntire S. L. (2009). Ethanol preference in *C. elegans* . Genes. Brain. Behav. 8, 578–585. 10.1111/J.1601-183X.2009.00513.X 19614755 PMC2880621

[B19] Markovinovic A. Greig J. Martín-Guerrero S. M. Salam S. Paillusson S. (2022). Endoplasmic reticulum-mitochondria signaling in neurons and neurodegenerative diseases. J. Cell Sci. 135, jcs248534. 10.1242/JCS.248534 35129196

[B20] Martinez B. A. Caldwell K. A. Caldwell G. A. (2017). *C. elegans* as a model system to accelerate discovery for Parkinson disease. Curr. Opin. Genet. Dev. 44, 102–109. 10.1016/J.GDE.2017.02.011 28242493

[B21] Moltedo O. Remondelli P. Amodio G. (2019). The mitochondria-endoplasmic reticulum contacts and their critical role in aging and age-associated diseases. Front. Cell Dev. Biol. 7, 172. 10.3389/fcell.2019.00172 31497601 PMC6712070

[B22] Moon Y. Lee K. H. Park J. H. Geum D. Kim K. (2005). Mitochondrial membrane depolarization and the selective death of dopaminergic neurons by rotenone: Protective effect of coenzyme Q10. J. Neurochem. 93, 1199–1208. 10.1111/J.1471-4159.2005.03112.X 15934940

[B23] Nussbaum-Krammer C. I. Neto M. F. Brielmann R. M. Pedersen J. S. Morimoto R. I. (2015). Investigating the spreading and toxicity of prion-like proteins using the metazoan model organism <em>*C. elegans*</em>. J. Vis. Exp. 95, e52321. 10.3791/52321 PMC4354510 25591151

[B24] Obeso J. A. Rodriguez-Oroz M. C. Goetz C. G. Marin C. Kordower J. H. Rodriguez M. (2010). Missing pieces in the Parkinson’s disease puzzle. Nat. Med. 16, 653–661. 10.1038/NM.2165 20495568

[B25] Pagliaro L. Marchesini M. Roti G. (2021). Targeting oncogenic Notch signaling with SERCA inhibitors. J. Hematol. Oncol. 14, 8. 10.1186/S13045-020-01015-9 33407740 PMC7789735

[B26] Peterková L. Kmoníčková E. Ruml T. Rimpelová S. (2020). Sarco/endoplasmic reticulum calcium ATPase inhibitors: Beyond anticancer perspective. J. Med. Chem. 63, 1937–1963. 10.1021/ACS.JMEDCHEM.9B01509 32030976

[B27] Poewe W. Seppi K. Tanner C. M. Halliday G. M. Brundin P. Volkmann J. (2017). Parkinson disease. Nat. Rev. Dis. Prim. 3, 17013–17021. 10.1038/NRDP.2017.13 28332488

[B28] Rossi A. Pizzo P. Filadi R. (2019). Calcium, mitochondria and cell metabolism: A functional triangle in bioenergetics. Biochim. Biophys. acta. Mol. Cell Res. 1866, 1068–1078. 10.1016/J.BBAMCR.2018.10.016 30982525

[B29] Sanders L. H. Greenamyren J. T. (2013). Oxidative damage to macromolecules in human Parkinson disease and the rotenone model. Free Radic. Biol. Med. 62, 111–120. 10.1016/J.FREERADBIOMED.2013.01.003 23328732 PMC3677955

[B30] Sherer T. B. Betarbet R. Testa C. M. Seo B. B. Richardson J. R. Kim J. H. (2003). Mechanism of toxicity in rotenone models of Parkinson’s disease. J. Neurosci. 23, 10756–10764. 10.1523/JNEUROSCI.23-34-10756.2003 14645467 PMC6740985

[B31] Stiernagle T. (2006). Maintenance of *C. elegans* (february 11, 2006), WormBook. New York, United States: C. elegans Res. Community, Wormb, 1–11. 10.1895/wormbook.1.101.1 PMC4781397 18050451

[B32] Tadini-Buoninsegni F. Smeazzetto S. Gualdani R. Moncelli M. R. (2018). Drug interactions with the Ca2+-ATPase from sarco(Endo)Plasmic reticulum (SERCA). Front. Mol. Biosci. 5, 36. 10.3389/FMOLB.2018.00036 29696147 PMC5904271

[B33] Thirugnanam T. Santhakumar K. (2022). Chemically induced models of Parkinson’s disease. Comp. Biochem. Physiol. C. Toxicol. Pharmacol. 252, 109213. 10.1016/J.CBPC.2021.109213 34673252

[B34] Vrijsen S. Besora-Casals L. Van Veen S. Zielich J. Van Den Haute C. Hamouda N. N. (2020). ATP13A2-mediated endo-lysosomal polyamine export counters mitochondrial oxidative stress. Proc. Natl. Acad. Sci. U. S. A. 117, 31198–31207. 10.1073/pnas.1922342117 33229544 PMC7733819

[B35] Wu S. Lei L. Song Y. Liu M. Lu S. Lou D. (2018). Mutation of hop-1 and pink-1 attenuates vulnerability of neurotoxicity in *C. elegans*: The role of mitochondria-associated membrane proteins in parkinsonism. Exp. Neurol. 309, 67–78. 10.1016/J.EXPNEUROL.2018.07.018 30076829 PMC6579610

[B36] Xu H. Van Remmen H. (2021). The SarcoEndoplasmic reticulum calcium ATPase (SERCA) pump: A potential target for intervention in aging and skeletal muscle pathologies. Skelet. Muscle 11, 25. 10.1186/S13395-021-00280-7 34772465 PMC8588740

[B37] Xu J. Minobe E. Kameyama M. (2022). Ca2+ dyshomeostasis links risk factors to neurodegeneration in Parkinson’s disease. Front. Cell. Neurosci. 16, 867385. 10.3389/FNCEL.2022.867385 35496903 PMC9050104

[B38] Zampese E. Surmeier D. J. (2020). Calcium, bioenergetics, and Parkinson’s disease. Cells 9, 2045. 10.3390/CELLS9092045 32911641 PMC7564460

[B39] Zeng X. S. Geng W. S. Jia J. J. (2018). Neurotoxin-induced animal models of Parkinson disease: Pathogenic mechanism and assessment. ASN Neuro 10, 1759091418777438–15. 10.1177/1759091418777438 29809058 PMC5977437

[B40] Zhang J. Zhang L. Wu Z. Zhang P. Liu R. Chang M. (2021). The dopaminergic neuroprotective effects of different phytosterols identified in rice bran and rice bran oil. Food Funct. 12, 10538–10549. 10.1039/D1FO01509E 34570129

[B41] Zhou S. Wang Z. Klaunig J. E. (2013). *Caenorhabditis elegans* neuron degeneration and mitochondrial suppression caused by selected environmental chemicals. Int. J. Biochem. Mol. Biol. 4, 191–200. 24380023 PMC3867705

[B42] Zucchi R. Ronca F. Ronca-Testoni S. (2001). Modulation of sarcoplasmic reticulum function: A new strategy in cardioprotection? Pharmacol. Ther. 89, 47–65. 10.1016/S0163-7258(00)00103-0 11316513

