# Supplementary material for: SERCA inhibition improves lifespan and healthspan in a chemical model of Parkinson disease in Caenorhabditis elegans
Source: Front Pharmacol. 2023 May 22;14:1182428. doi: 10.3389/fphar.2023.1182428 (PMC10239880; doi:10.3389/fphar.2023.1182428)
Supplement: Supplementary file 1 [file DataSheet1.PDF]

Supplementary Material

SERCA inhibition improves lifespan and healthspan in a chemical model of Parkinson disease in C. elegans

Silvia Romero-Sanz, Elena Caldero-Escudero, Pilar Álvarez-Illera, Jaime Santo-Domingo,

Rosalba I Fonteriz, Mayte Montero, and Javier Álvarez*

*** Correspondence:** Javier Alvarez: javier.alvarez.martin@uva.es

Supplementary Tables 1 and 2, and Figures S1 and S2

Condition	$T_{½}$ (days)	n (final/total)	% $T_{½}$ change vs control	% $T_{½}$ change vs Rot
N2	16.7	71/81		
Rotenone	12.7	69/85	↓24.0% **	
Rot+*sca-1* RNAi	18.9	51/83		↑48,9% ***
N2	15.7	99/127		
Rotenone	9.9	115/125	↓37.0% **	
Rot+*sca-1* RNAi	12.5	102/117		↑26,3% ***
N2	15.8	97/111		
Rotenone	9.9	80/105	↓ 37,3% ***	
Rot+*sca-1* RNAi	13.7	70/99		↑38,4 % **
SJ4103	15.9	97/108		
Rotenone	12.1	93/97	↓23,9% **	
Rot+*sca-1* RNAi	17.1	67/67		↑41.3% ***
SJ4103	22.2	68/81		
Rotenone	13.7	71/75	↓38.3% **	
Rot+*sca-1* RNAi	15.4	69/74		↑12.4% *
SJ4103	18.1	83/109		
Rotenone	8.2	54/64	↓54.5% ***	
Rot+*sca-1* RNAi	10.5	95/131		↑28,0% *

Table S1. The tables shows the half-life in days (T½) of the worms obtained from the Kaplan-Meier analysis, the number of worms in each assay (n final/total), the % half-life change and the significance of the difference obtained from the log-rank test. **, $p<0.01$; ***, $p<0.005$.

Parameter	Condition	Mean ± s.e.m. (1)	Mean ± s.e.m. (2)	n1/n2	Significance
Average Speed (mm/s)	N2 vs Rot	0.116 ± 0.007	0.045 ± 0.005	53/54	$p<0.0001$
	N2 vs Rot + *sca-1*	0.116 ± 0.007	0.068 ± 0.004	53/97	$p<0.0001$
	Rot vs Rot + *sca-1*	0.045 ± 0.005	0.068 ± 0.004	54/97	$p<0.005$
Maximum Speed (mm/s)	N2 vs Rot	0.241 ± 0.009	0.139 ± 0.011	53/54	$p<0.0001$
	N2 vs Rot + *sca-1*	0.241 ± 0.009	0.180 ± 0.008	53/97	$p<0.0001$
	Rot vs Rot + *sca-1*	0.139 ± 0.011	0.180 ± 0.008	54/97	$p<0.005$
Area (mm^2)	N2 vs Rot	0.145 ± 0.004	0.086 ± 0.003	53/54	$p<0.0001$
	N2 vs Rot + *sca-1*	0.145 ± 0.004	0.086 ± 0.002	53/97	$p<0.0001$
	Rot vs Rot + *sca-1*	0.086 ± 0.003	0.086 ± 0.002	54/97	n.s.
BLPS	N2 vs Rot	0.075 ± 0.005	0.041 ± 0.004	53/54	$p<0.0001$
	N2 vs Rot + *sca-1*	0.075 ± 0.005	0.061 ± 0.004	53/97	$p<0.05$
	Rot vs Rot + *sca-1*	0.041 ± 0.004	0.061 ± 0.004	54/97	$p<0.005$
BBPS	N2 vs Rot	0.141 ± 0.009	0.090 ± 0.011	53/54	$p<0.001$
	N2 vs Rot + *sca-1*	0.141 ± 0.009	0.112 ± 0.007	53/97	n.s.
	Rot vs Rot + *sca-1*	0.090 ± 0.011	0.112 ± 0.007	54/97	n.s.
Defecation rate	N2 vs Rot	28.55 ± 1.11	7.27 ± 1.48	11/11	$p<0.0001$
	N2 vs Rot + *sca-1*	28.55 ± 1.11	13.00 ± 1.75	11/10	$p<0.0001$
	Rot vs Rot + *sca-1*	7.27 ± 1.48	13.00 ± 1.75	11/10	$p<0.05$
Pumping rate	N2 vs *sca-1*	277 ± 5	290 ± 10	15/20	n.s.
	N2 vs Rot	277 ± 5	17.7 ± 3.0	15/13	$p<0.0001$
	Rot vs Rot + *sca-1*	17.7 ± 3.0	32.2 ± 4.6	13/11	$p<0.05$

Table S2. The table shows the mean data and statistical significance of the measurements of mobility, defecation and pumping in the different conditions. n is the number of worms analyzed in each condition.

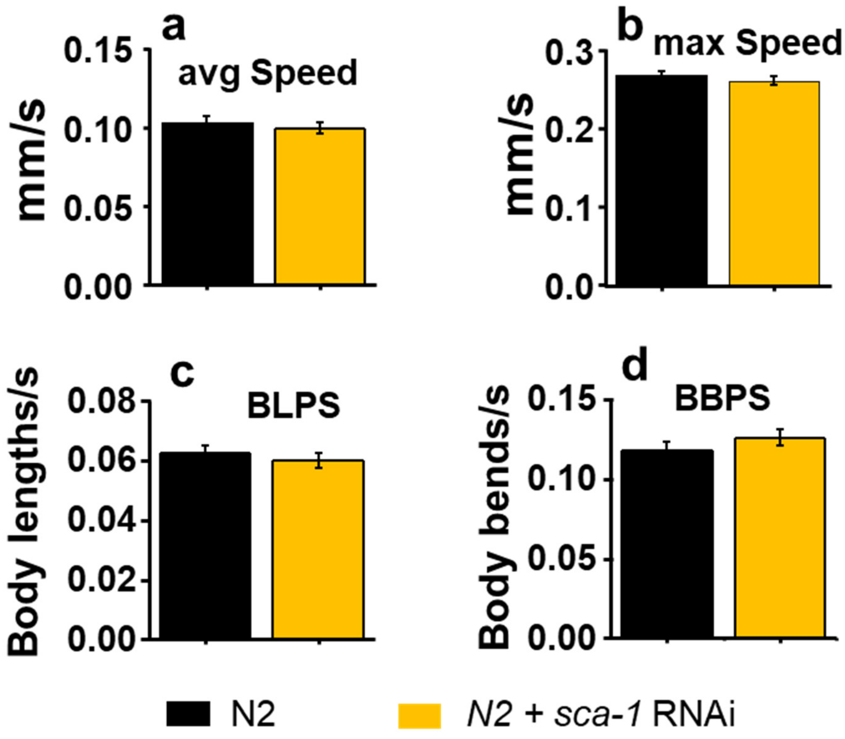

Figure S1

Figure S1. Effect of *sca-1* RNAi on N2 *C. elegans* mobility. The figure shows the average speed (panel a), maximum speed (panel b), speed measured as body length per second (BLPS, panel c) and body bends per second (panel d) in either control worms or worms treated with 10% *sca-1* RNAi. Data are mean ± s.e.m. No significant differences were obtained.

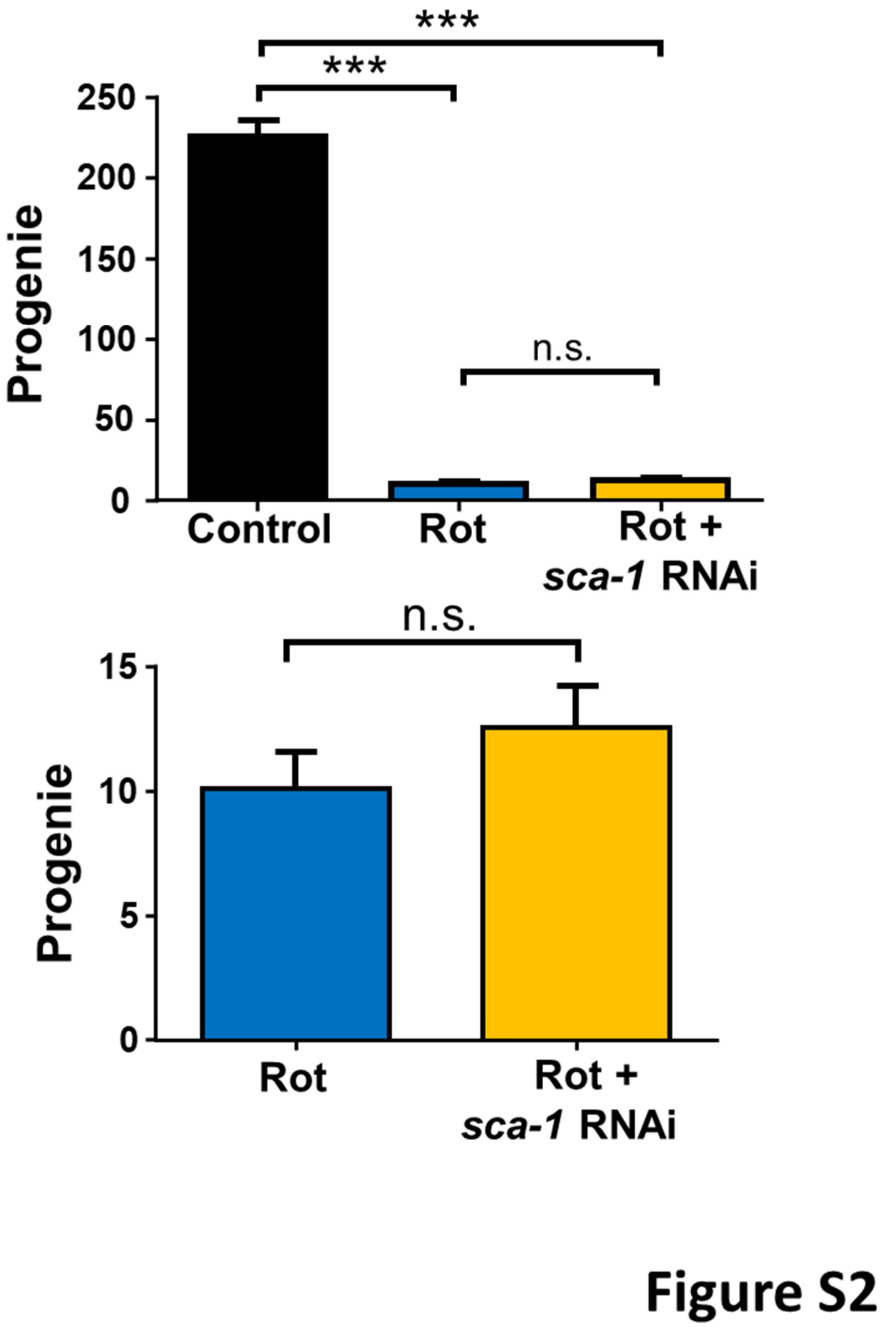

Figure S2. Effect of rotenone and *sca-1* RNAi on the fertility of the *C. elegans* N2 strain. ***, $p<0.005$; ANOVA test.
